# The interplay of HIV and human papillomavirus-related cancers in sub-Saharan Africa: scoping review

**DOI:** 10.1186/s13643-020-01354-1

**Published:** 2020-04-22

**Authors:** Kabelo Matjie Bridget Lekoane, Desmond Kuupiel, Tivani P. Mashamba-Thompson, Themba G. Ginindza

**Affiliations:** 1 Discipline of Public Health Medicine, School of Nursing and Public Health, University of KwaZulu-Natal, Durban, South Africa; 2 Department of Public Health Medicine, School of Nursing and Public Health, University of KwaZulu-Natal, Durban, South Africa

**Keywords:** HPV, HPV-related cancers, HIV, Sub-Saharan Africa

## Abstract

**Background:**

People living with HIV (PLHIV) are at a high risk of developing HPV-related cancers. HPV-related malignancies occur frequently and/or are high among PLHIV, with cervical cancer as a designated AIDS-defining condition. We aimed to explore the evidence on the interplay of HIV and HPV-related cancers in sub-Saharan Africa (SSA).

**Methods:**

The scoping review was guided by Arksey and O’Malley’s framework. We searched for literature from the following databases: PubMed; World Health Organization (WHO) Library; Science Direct; Google Scholar and EBSCOhost (Academic search complete, Health Source: Nursing/Academic Edition, CINAHL). Studies reporting on evidence HIV and HPV-related cancers interplay in SSA were eligible for inclusion in this review. The Mixed Methods Appraisal Tool (MMAT) tool was used to assess the risk of bias of the included studies. Preferred Reporting Items for Systematic Reviews and Meta-Analyses (PRISMA) was used for reporting the search results. Thematic analysis used to reveal the emerging themes from the included studies.

**Results:**

A total of 74 potentially eligible articles were screened. Of these, nine (7 reviews, 1 transversal case controls, and 1 quantitative study) were eligible for data extraction. The studies reported about a total of 16,351 participants in different settings. The nine included studies showed evidence of cervical cancer among HIV-infected women and distribution of HPV infection and cervical abnormalities among HIV-positive individuals. In the four studies generalizing about HIV and anal cancer, only one reported about HPV. Two studies generally reported about HIV and head and neck cancers and one reported about interaction of HIV with vaginal cancer, vulvar cancer, and penile cancer, respectively.

**Conclusion:**

HIV positivity is associated with increased prevalence of HPV infection on different anatomic sites, which will result in increased burden of HPV-related cancers among PLHIV. Furthermore, primary studies with robust study designs aimed at investigating the risk developing HPV-related cancers among PLHIV are recommended.

Systematic review registration: PROSPERO CRD42017062403

## Background

The International Agency for Research on Cancer (IARC) classified both human papillomavirus (HPV) and human immunodeficiency virus (HIV) type 1 as carcinogens [1], and studies demonstrated that HPV is a direct carcinogen and HIV-1 is an indirect carcinogen through immune suppression [1]. HPV infection is etiologically responsible for all cervical cancers, a subset of cancers of the anus, oropharynx, penis, vagina, and vulva [2, 3]. Individuals with HIV and acquired immunodeficiency syndrome (AIDS) are at a high risk of developing HPV-related cancers [4–9]. Incidence of HPV-related diseases continued to rise as compared to the incidence of many HIV-associated comorbidities, which decreased in the era of antiretroviral therapy (ART) [10, 11]. According to the United Nations Programme on AIDS (UNAIDS), in 2016, the clear majority of PLHIV were from low- and middle-income countries (LMIC) with 25.5 million located in SSA [12]. With the aging of the population of HIV-infected individuals due to combined antiretroviral therapy (cART), the landscape of malignancies has shifted. Classical AIDS-defining malignancies (ADM) have decreased; however, other non-AIDS-defining malignancies (NADMs) such as anal, lung, colorectal, and liver cancers continue to pose a significant health threat to patients with HIV [13, 14].

As evidenced by the HIV and AIDS Cancer Match Study, cervical cancer incidence among HIV-infected women in the USA has been unchanged since the introduction of ART at 64.2 cases per 100,000 person-years from 1990 to 1995 compared to 86.5 cases per 100,000 person-years from 1996 to 2002 [15]. From cohort studies it was reported that anal cancer rates appear to be increasing and the anal cancer incidence in men with HIV has climbed from 35 to 49 cases per 100,000 person-years in the pre-ART era to the range of 92 to 144 cases per 100,000 person-years [16, 17]. The risk of cancer in HIV-infected individuals as compared to the general population increased as high as 29 times for anal cancer, 4 times for penile cancer, 6 times for vulvar and vaginal cancer as well as for cervical cancer [1].

The introduction of HPV vaccine in the immunization programs in high-income countries had played a key role in the reduction of the incidence of cervical cancer using either the quadrivalent (*Gardasil*) or the bivalent (*Cervarix*) HPV vaccine [18]. HPV Quadrivalent Gardasil vaccine was introduced, and it provides protection against persistent infection and disease associated with HPV types 6, 11, 16, and 18 for women with no evidence of prior HPV infection and became FDA-approved in 2006 [18]. Studies demonstrated that HPV vaccination prevented the infection of HPV types 16 and 18 infections and associated cervical epithelial neoplasm 2 or 3, adenocarcinoma in situ, or invasive cervical cancer with 98% efficacy in young women without prior HPV 16 or 18 infections [18]. Efficacy in preventing the development of genital warts and vulvar, vaginal, and perianal HPV-related disease associated with the vaccine HPV types in women without prior infection with these HPV types were reported to be 100% [19]. Although there are limited treatment options for HPV-associated diseases in HIV-infected people, there are opportunities for disease prevention with vaccination. HPV-associated cancers in HIV-infected persons persist irrespective of the apparent immune reconstitution with ART [20] and the importance of optimizing screening; prevention and treatment of these diseases become even more important.

Therefore, the aim of this study is to explore the evidence on the interplay of HIV and HPV-related cancers in SSA. It is anticipated that the study findings will help to strengthen the implementation of control and prevention policy and the planning of prevention strategies incorporating HPV infection prevention especially among the youth and HIV-infected people. Even though there is information about HPV vaccine and testing within cervical cancer screening programs, it has been shown that the patients’ and public’s knowledge on HPV-related cancers is still limited [21, 22]. Therefore, contributions of a scoping review gains importance and relevance by demonstrating the current evidence to identify research gaps and suggest novel ideas for future research.

## Methods

### Study design

This study is a part of the larger study entitled “Mapping evidence on the distribution of human papillomavirus-related cancers in sub-Saharan Africa: scoping review protocol.” This large study is registered in PROSPERO under registration number CRD42017062403. It is accessible via this link: https://www.crd.york.ac.uk/PROSPERO/display_record.asp?ID=CRD42017062403.

A comprehensive description of the methods used in this study can be found in the published protocol [23].

Scoping review was chosen as the best method to map the evidence on interplay of HIV and HPV-related cancers in SSA. Arksey and O’Malley’s framework [24] incorporating the recommendations of Levac et al. [25] was employed to guide this scoping review study. The Arksey and O’Malley framework involves identifying the research question; identifying the relevant literature; identifying the study selection; charting the data; and collating, summarizing, and reporting the results. We searched for peer-reviewed quantitative studies reporting on the interplay of HIV and HPV-related cancers in SSA. We used Pluye et al.’s version 2011 tool to perform quality assessment of the included primary studies [26]. The results of this scoping review are presented according to the recommendations of Preferred Reporting Items for Systematic Reviews and Meta-Analyses (PRISMA) [27]. We applied the PEOs (population, exposure, outcome, and setting) framework for scoping reviews in determining the eligibility research question for a scoping review project.

### Search strategy

We searched several online databases including PubMed, World Health Organization (WHO) Library, Science Direct, and Google Scholar, and within EBSCOhost platform, the following databases were searched: Academic Search Complete, Health Source: Nursing/Academic Edition, and CINAHL with full text for published studies. The search took place from April 2017 to July 2017. We used the following keywords on the search databases: HPV-related cancers, HIV, prevalence, incidence, and sub-Saharan Africa (to search titles of the eligible studies). Boolean terms AND/OR were used to separate the keywords during the search. There was no language and date restriction. Medical Subject Headings (MeSH) terms were included in the search. The literature search strategy in the databases was developed by KMBL in consultation with a librarian. Reference sections of included full articles were reviewed to identify additional eligible articles. Detailed description of the database search strategy is attached in Additional file 1: Table S1.

### Study selection

Firstly, KMBL searched the eligible titles guided by the eligibility criteria from the databases and imported them to a new Endnotes X7 library created for this scoping review. Duplicates were removed before the start of the abstract screening phase. Both abstract and full article screening tool were developed in Google forms using the inclusion criteria. Secondly, two skilled reviewers (KMBL and DK) screened all retrieved abstracts against the eligibility criteria. To be selected for the next stage of the review, a study had to be conducted among humans and report of data on HIV and assessment of the interplay with HPV-related cancers are needed. If the reviewer was uncertain of the eligibility of the study population while the intervention, outcome, and study setting were eligible, the article was not excluded but rather carried onto the next stage of the selection process. Disagreement at this stage were resolved via discussions until a consensus was reached. Again, two independent reviewers (KMBL and DK) did a full article screening and a third reviewer (TPM-T) was involved to resolve the disagreements. To measure the inter-rater agreement between reviewers, we used Cohen’s kappa coefficient (κ) statistic using Stata 13.0SE (StataCorp College Station, TX, USA).

### Eligibility criteria

### Inclusion criteria

This study included the following:

- Studies involving individuals of all ages
- Studies focused on HIV and HPV-related cancers
- Studies reporting evidence from SSA countries (Angola, Benin, Botswana, Burkina Faso, Burundi, Cameroon, Cape Verde, Central African Republic, Chad, Comoros, Congo (Brazzaville), Congo (Democratic Republic Côte d'Ivoire, Djibouti, Equatorial Guinea, Eritrea, Ethiopia, Gabon, The Gambia, Ghana, Guinea, Guinea-Bissau, Kenya, Lesotho, Liberia, Madagascar, Malawi, Mali, Mauritania, Mauritius, Mozambique, Namibia, Niger, Nigeria, Réunion, Rwanda, Sao Tome and Principe, Senegal Seychelles, Sierra Leone, Somalia, South Africa, Sudan, Swaziland, Tanzania, Togo, Uganda, Western Sahara, Zambia, Zimbabwe).
- Quantitative study designs

### Exclusion criteria

This study excluded the following:

- Studies that do not focus on HPV-related cancers
- Studies that did not focus on HIV and HPV-related cancers
- Studies that reported about HIV only
- Studies that reported evidence from countries outside SSA

### Charting data

We abstracted the data from the included studies using a piloted form designed in Google forms. The following information was extracted from included studies: author and date, study setting, intervention, age, percentage of males/females, gender, study aim, and study designs.

### Summary and collating

Thematic analysis was performed to identify the interplay of HIV and HPV-related cancers in SSA from the included studies. The included manuscripts were manually coded into categories, which were grouped into the following five themes:

- Cervical cancer
- Anal cancer
- Vaginal and vulvar cancers
- Head and neck cancers
- Penile cancer

### Quality of evidence

The quality assessment of included studies was performed using Mixed Methods Appraisal Tool (MMAT)–Version 2011, with the purpose of evaluating the risk of bias [26]. Two reviewers (KMBL and DK) independently assessed the quality of the studies included and the results compared for accuracy and consistency, and TMP-T resolved the discrepancies. The risk of bias scale score for studies was based on the following domains: clarity of the research questions, confidence in the assessment of the research question, appropriateness of data sources collected; and suitability of statistical analysis to address the research question or objective. There are other domains included: clear description of the design and allocated concealment, complete outcome data and the withdrawal, recruitment of participants, appropriate measurements and comparable participants, relevant sample and representative of the population, relevant design and integration of the methods, and researcher’s appropriate consideration of the method. An overall quality percentage score of the included studies was calculated, and the scores were interpreted as high quality (76–100%), average quality (51 to < 76%), and low quality (≤ 50%).

## Results

**Fig. 1:**
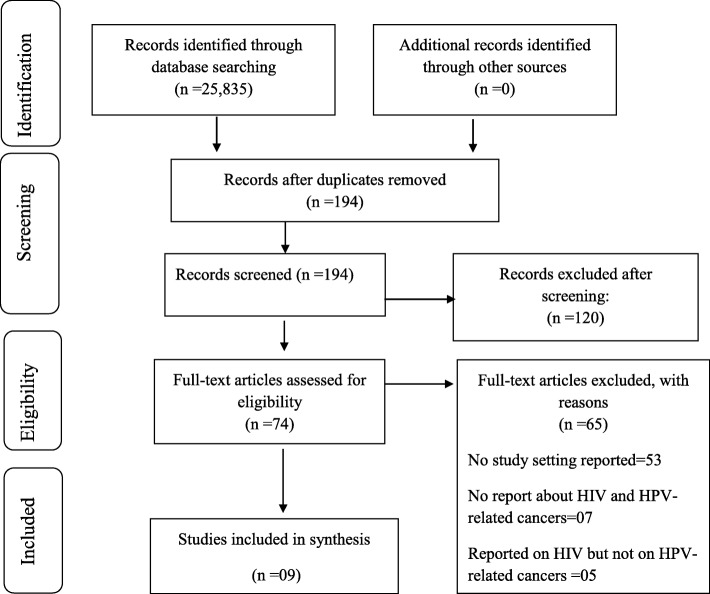
PRISMA chart showing literature search and selection of studies

The initial search yielded a total of 25,835 articles from all databases. Applying our exclusion criteria, the number of studies reduced to 194 (Fig. 1). The abstract screening tool was used, and the eligible 194 articles were screened against the eligibility criteria, from those 74 which were included for full article screening. From the 74 studies screened at full article screening, 9 articles were deemed eligible for inclusion in data extraction. The inter-rater agreement at the full-text screening phase was 86.49% versus 64.57% expected by chance which constitutes moderate to substantial agreement (Kappa statistic = 0.62, *p* value < 0.05). In addition, McNemar’s chi-square statistic suggests that there is not a statistically significant difference in the proportions of yes/no answers by the reviewer with a *p* value > 0.05 (Additional file 2: Table S1).

A total of 120 studies were excluded from this study as they did not meet the inclusion criteria for this study. Of those 74 studies that underwent full manuscript review, 65 of them were found to have no valuable data for analysis in this study. A total of 53 articles did not report anything about the study setting of interest which is SSA [1, 28–79], 7 did not report anything about HIV and HPV-related cancers [2, 41, 80–84], and 5 reported about HIV but not HPV-related cancers [85–89].

### Characteristics of the included studies

The included studies comprise of review articles which made the bulk of the articles with 77.7% (7/9) [90–96] and (1/9) transversal case control study [97] and (1/9) quantitative study [98] and each contributed 11.1% respectively.

**Fig. 2:**
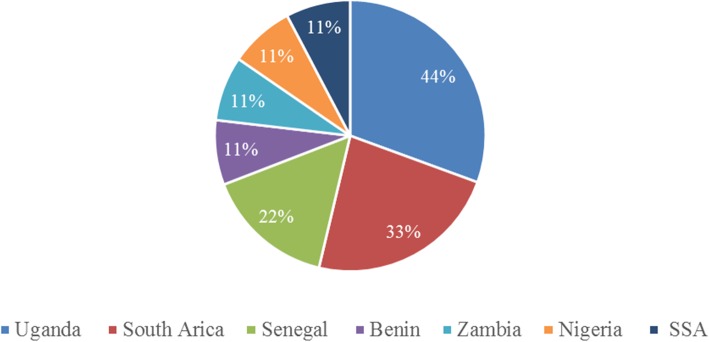
Showing the countries reported in the included studies (*N* = 9)

The studies were done and reviewed from different study settings. About 11.1% (1/9) study generalized about SSA with no specific country mentioned [90], 44% (4/9) reported about Uganda [91, 92, 95, 96], 33% (3/9) South Africa (SA) [93, 95, 96], 22% (2/9) Senegal [94, 96], (1/9) Zambia [93], (1/9) Nigeria [98], and (1/9) Benin [97], and each contributed 11% respectively. Among the included studies, only three were conducted in an urban setting; those of the rest of the studies were not mentioned. The countries which were reported in the included studies are presented in Fig. 2.

**Fig. 3:**
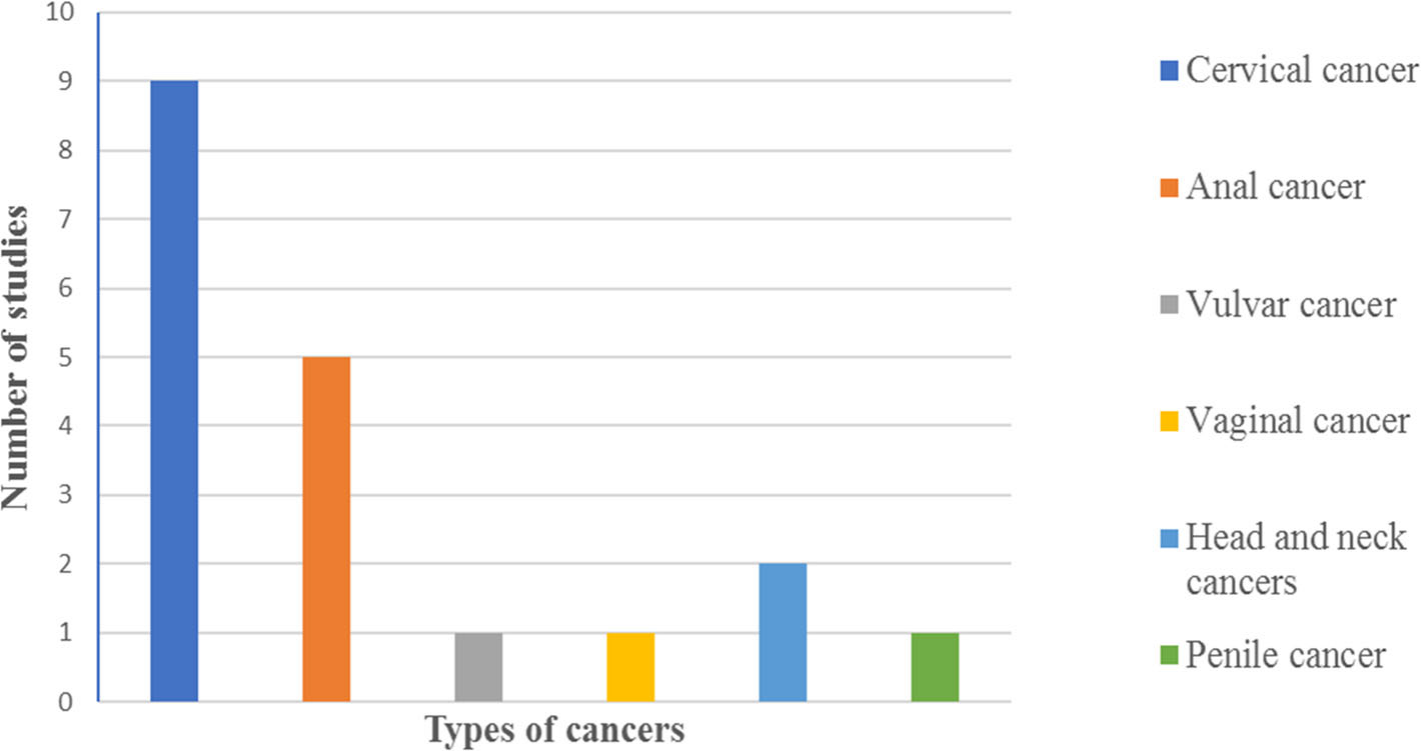
Showing the number of included studies reported on different cancers in the study (*N* = 9)

The nine included studies showed evidence of cervical cancer among HIV-infected women and distribution of HPV infection and cervical abnormalities among HIV-positive individuals [90–98]. Five studies generalized about HIV and anal cancer [92–94, 96, 98]; only one reported about HIV and penile cancer [93]. Two studies [93, 98] generally reported about HIV and its association with head and neck cancers, and one [93] reported about vaginal cancer, vulvar cancer, and penile cancer. Figure 3 presents the number of included studies that reported on different anatomic cancer sites.

All included studies predominantly reported on women, followed by men and man having sex with man (MSM), except for one which generalized on children and adolescents [90]. The age of the population was not included in most studies, and only one study focused on women of 20 to 60 years old with an average age of 38 years [97]. The total number of participants from all the included studies was 16,351. The characteristics of the included studies are presented on Table 1.

**Table 1:** Characteristics of the included studies

Author and date	Study population			Study setting	Geographical location	Type of cancer indicated	Study design	Outcome
	Gender and age Number or population percentage type							
Bosch, 2013 [90]	Women, children, men	Not specified	Not specified	SSA	Not specified	Cervical cancer, anal cancer	Review	HIV-positive women had two- to 22-folds increase risk of developing cervical cancer as compared to HIV-negative women
Brickman, 2015 [91]	Female, male	Not specified	15,000 HIV-infected individuals	SSA Uganda	Kyadondo County	Cervical anal cancer	Review	Incidence of cervical cancer was reported to be 70 per 100,000 women-years and a SIR of 2.7 among HIV-infected women as compared to HIV-uninfected women
Capo-chichi, 2016 [97]	Women (sexually active)	20 to 60 years old (average 38 years)	86 = control group 86 = HIV-infected women	Benin	Contonou	Cervical cancer	Transversal case control	HIV-infected women have a higher prevalence and persistence of HPV infection, an increased risk for abnormal Pap smears and cervical cancer -The study further highlighted that among the HIV-negative group, one woman with HPV and no lamin A/C developed cervical lesions and two women with no HPV but with total absence of lamin A/C developed cervical cancer -Five had low-grade squamous intraepithelial0 lesion (LSIL), one had high-grade squamous intraepithelial lesion (HSIL), and 4/86 (5%) had cervical dysplasia among HIV-infected women
De Vuyst, 2013 [92]	Women Men	Not specified	Not specified	SSA	Not specified	Cervical cancer, anal cancer	Review	-HIV positivity is associated with increased prevalence of cervical HPV infection -HIV-infected individuals who live longer on cART may be at increased risk of persistent HPV infection and precancerous CIN progressing to cervical cancers
			379 HIV-negative and 107 HIV-positive women with ICC	Uganda				
Firnhaber, 2012 [93]	Women Men MSM	Not specified	400	South Africa	Cape Town	Cervical cancer Anal cancer Oropharyngeal cancer Vaginal cancer Vulvar cancer	Review	-HIV-infected women have a higher prevalence of HPV infection and cervical abnormalities compared to HIV-seronegative women throughout the world -Studies confirmed that HIV-positive women harbored high-risk HPV -HPV-related anal cancer is increasing in both HIV-infected women and men despite ARV treatment -Vulvar cancer occurs in HIV-infected women who are less than 40 years -HIV-infected people appear to have a rate of two to four times higher of oropharyngeal cancer than HIV-uninfected populations -The risk of invasive penile cancer is estimated to be 20 times higher among HIV-infected men as compared to HIV-uninfected men
			145	Zambia				
			148	South Africa SSA				
Heard, 2011 [94]	Women	Not specified	Not specified	SSA Senegal	Not specified	Cervical cancer	Review	Study from Senegal reported a substantial increase in the risk of invasive cervical cancer (ICC) which was observed with OR of 6.5 for ICC (95% CI 2.1–19.8) in HIV-infected women compared with control group of HIV-negative women
Jedy-Agba, 2016 [98]	Female, male	Not specified	Not specified	SSA Nigeria	Not specified	Cervical cancer Oropharyngeal cancer Anal cancer	Quantitative	Study in Nigeria reported a two-fold higher risk of cervical cancer in people living with HIV/AIDS
Louie, 2009 [95]	Women	Not specified	Not specified	South Africa Uganda	Not specified	Invasive cervical cancer	Review	HIV-infected women are at higher risk of being infected with Hr-HPV and are at a higher risk for persistence and associated cervical disease progression than HIV-uninfected women
Palefsky, 2006 [96]	Men who have sex with men Women	Not specified	Not specified	SSA South Africa Uganda Senegal	Not specified	Anal cancer Cervical cancer	Review	HIV positivity is associated with increased prevalence of cervical HPV infection and cervical intraepithelial neoplasia

A total of 120 studies were excluded from this study as they did not meet the inclusion criteria for this study. Of those 74 studies that underwent full manuscript review, 65 of them were found to have no valuable data for analysis in this study. A total of 53 articles did not report anything about study setting of interest which is SSA [1, 26–79], 7 did not report anything about HIV and HPV-related cancers [2, 41, 79–83], and 5 reported about HIV but not HPV-related cancers [85–89].

### Quality of evidence from included primary studies

From the included ten studies, two primary studies underwent methodological quality assessment using the appropriate section of the Mixed Methods Appraisal Tool (MMAT)–Version 201 1[25]. Both studies scored 100% [97, 98] (Additional file 3: Table S3). The remaining eight included studies were not appraised for quality because they were not primary studies.

### Findings of the studies

The scoping review revealed findings about HPV-related cancer and their interplay with HIV. These cancers are cervical cancer, anal cancer, vulvar and vaginal cancers, head and neck cancers, and penile cancer.

#### Cervical cancer

The nine included studies reported about cervical cancer, HPV, and HIV [90–98]. The number of women living with HIV ranges was estimated to be less than 1000 cases in Comoros to 3.2 million in South Africa [95], whereas according to Bosch et al., HIV-positive women had two- to 22-folds increase risk of developing cervical cancer as compared to HIV-negative women [90]. Two studies demonstrated a 2.4 standard incidence ratio (SIR) of cervical cancer in HIV-positive women from Uganda AIDS–Cancer Registry Match Study [99], and the second from Senegal showed a substantial increase in the risk of invasive cervical cancer (ICC) which was observed with OR of 6.5 for ICC (95% CI 2.1–19.8) in HIV-infected women compared with the control group of HIV-negative women [100]. The incidence of cervical cancer in a linkage of 15,000 HIV-infected individuals from Kyadondo County in Uganda reported 70 per 100,000 women-years and an SIR of 2.7 among HIV-infected women as compared to HIV-uninfected women [91]. Another study in Nigeria reported a two-fold higher risk of cervical cancer in people living with HIV/AIDS [97]. Two recent reviews have shown that HIV positivity is associated with increased prevalence of cervical HPV infection [92, 96] and cervical intraepithelial neoplasia (CIN) [96]. Furthermore, the study demonstrated a higher number of CIN among South African HIV-positive women as compared to HIV-negative women [96].

Recent review performed by Palesky has shown that HIV positivity is associated with increased prevalence of cervical HPV infection and cervical intraepithelial neoplasia (CIN) [96]. Furthermore, the study demonstrated a higher number of CIN among South African HIV-positive women as compared to HIV-negative women [96]. It has been reported that HIV-infected women have a higher prevalence and persistence of HPV infection, an increased risk for abnormal Pap smears and cervical cancer [97]. It was also confirmed by a transversal case control study conducted in Benin, aimed at verifying the HPV genotypes among women living with HIV and treated with ARV compared to women with no HIV infection that focus on HIV and HPV-related cancers [97]. In the study, only 5 had low-grade squamous intraepithelial lesion (LSIL), one had high-grade squamous intraepithelial lesion (HSIL), and 4/86 (5%) had cervical dysplasia among HIV-infected women. However, high-risk HPV (HR-HPV) counts was higher among groups with HIV infections (*n* = 43) compared to the HIV-negative group. Additionally, Hr-HPV infection was significantly high (27/86 = 31%) in HIV-positive women compared to HIV-negative women (20/86 = 23%). The study further highlighted that among the HIV-negative group, one woman with HPV and no lamin A/C developed cervical lesions and two women with no HPV but with total absence of lamin A/C developed cervical lesions [96]. A review by Louie et al. also reported that HIV-infected women are at a higher risk of being infected with Hr-HPV and are at a higher risk for persistence and associated cervical disease progression than HIV-uninfected women [95].

A review by Firnhaber et al. showed that HIV-infected women have a higher prevalence of HPV infection and cervical abnormalities compared to HIV-seronegative women throughout the world [92], and this was confirmed by three studies [101–103]. The first study was conducted in Cape Town, South Africa, among 400 untreated HIV-1-infected women who underwent HPV and cervical screening; the results showed 68% of women were positive for HR HPV DNA types and 35 and 13% of these women had LSIL and HSIL, respectively, on Papanicolaou (Pap) smears [101]. The second study conducted in Zambia among 145 women found that 98% of HIV-infected women harbored at least one type of HPV (85% had an HR HPV type), with a median of four types per participant; HPV type 52 was most the common at 37.2%, with types 16 (17.2%) and 18 (13.1%) present in far fewer women [101]. Evidenced by the third study conducted in South Africa, among 148 HIV-infected women showed comparable results, with 95% of HIV-infected women harboring HPV [103]. One multicenter study from Uganda found HPV distribution in 107 HIV-infected and 309 HIV-uninfected women with ICC [92]. It has been shown that HIV-infected individuals who live longer on cART may be at increased risk of persistent HPV infection and precancerous CIN progressing to cervical cancers [92]. Although there is evidence reported on the association of HIV, HPV, and cervical cancer, more studies should be conducted to investigate the contribution of HPV in cervical cancer cases among HIV-infected women. Again, more studies to investigate the distinguishable effect of ART among HIV-infected women are required. Furthermore, studies to investigate to what extent does HIV confers to the development of cervical HPV attributable cancer in HIV-infected women are recommended.

#### Anal cancer

Five of the included studies generally reported about anal cancer, HPV, and HIV [92–94, 96, 98]. One study showed that the knowledge of the prevalence of anal cancer in HIV-infected men and women in resource-limited countries is very limited, yet on the other hand, HPV-related anal cancer is increasing in both HIV-infected women and men despite ARV treatment [93]. Studies have demonstrated high prevalence of anal HPV infection, anal precancerous lesions, and anal cancer in HIV-positive individuals in both men and women [94]. Two studies have shown that anal HPV infection and anal intraepithelial neoplasia (AIN) are remarkably common among both HIV-positive and HIV-negative women and the incidence is elevated in both HIV-positive and HIV-negative MSM [96, 98]. A review published by De Vuyst et al. show that there is an increased risk of persistent HPV infection and precancerous AIN that progresses to anal cancer among HIV-infected individuals [92]. Despite the revealed findings, it is shown that more studies to determine the prevalence or burden of anal cancer among HIV-infected men, women, and MSM are missing. Further studies to determine whether the use of ART result in regression of anal HPV lesions in HIV-infected individuals are recommended.

#### Vulvar and vaginal cancers

One of the nine included studies reported about vulvar and vaginal cancers and HIV [92]. A report in the literature indicated that vulvar cancer occurs in HIV-infected women who are less than 40 years [93]. It is often seen in the post-menopausal stage in the 7th or 8th decade of life [94]. Vaginal cancer and vaginal HPV infection are also reported to be higher in HIV-infected patients than in HIV-seronegative women, and this cancer occurs at a younger age in HIV-infected individuals [93]. Further studies to assess the risk of acquiring vaginal and vulvar cancers among HIV-infected women are missing.

#### Head and neck cancers

Two from the included studies generalized about oropharyngeal cancer and HIV [93, 98]. It has been reported that other HPV-attributable cancers like oropharyngeal cancers have also been linked with HIV infection [98] and the HIV-infected people appear to have a rate of two to four times higher of oropharyngeal cancer than that of HIV-uninfected populations [93]. Although evidence between HIV and oropharyngeal cancer was identified, it was not sufficient to draw a conclusion on the interplay between HIV and HPV-related head and neck cancers as it reported on oropharyngeal cancers; instead, more studies are recommended.

#### Penile cancer

One of the nine included studies generalized about penile cancer and HIV [93]. According to the review published by Firnhaber et al., the risk of invasive penile cancer is estimated to be 20 times higher among HIV-infected men as compared to HIV-uninfected men [92]. Moreover, a second study estimated penile cancer risk to be four times higher in HIV-infected men [93]. More studies are still required on the interaction between HPV-related penile cancer and HIV; one study is insufficient.

## Discussion

The study was aimed at exploring the evidence on the interplay of HIV and human papillomavirus-related cancers in SSA. This scoping review provided a general overview and evidence on the interplay of HIV with cancer of the cervix, anus, vulvar, vaginal, penile, and head and neck in SSA. This scoping review presented evidence from six countries within SSA; only two studies were conducted in urban areas and not specified in most of the studies. Again, generally there is a lack of studies conducted specifically on males as opposed to those conducted on females. The findings demonstrated a gap in literature on HPV-related cancers among HIV-infected individuals in many countries within SSA and lack of research in rural areas and poor participation among men. Our results show that there is limited evidence to confirm the presence of HPV genotypes on different anatomic cancer sites among PLHIV. It also shows scarcity of studies aimed at evaluating the risk of developing HPV-related cancers among HIV-infected people. Although evidence on interplay of HIV between vulvar cancer, vaginal cancers, head and neck cancers, and penile cancer has been identified, it was not sufficient to make a generalization and conclusion due to the limited evidence.

This study shows that the prevalence of cervical HPV infection and CIN is increased with HIV positivity [96]. In agreement with our findings, cohort studies provided persuasive evidence that the risk of acquiring cervical HPV infection is higher in HIV-positive women as compared to HIV-negative women [104–108]. This scoping review shows in the study conducted in Benin that five of HIV-infected woman had LSIL and one had HSIL, respectively [97], and results from another study in South Africa reported 35% LSIL and 13% HSIL among HIV-infected women [101]. Similarly, the prevalence of LSIL and HSIL in women infected with HIV is reported as 28.6 and 14% in the UK [109], 15.4 and 7.9% in the USA [110], and again 21.0 and 2.8% in a European cohort [111]. Several studies from this scoping review shows that HIV-infected women harbor different Hr-HPV genotypes [97, 101–103]. In line with this study, persistent HPV types were shown to be 24% in HIV-positive women versus 4% in HIV-negative women [112] and again a broader range of both Hr-HPV and low-risk HPV types were found in HIV-positive women as compared to the HIV-negative women [113–115].

This study has been shown that knowledge on the prevalence of anal cancer in HIV-infected men and women in resource-limited countries is very limited, yet on the other hand, HPV-related anal cancer is increasing in both HIV-infected women and men despite ARV treatment [93]. In line with this, a study conducted to check the incidence and risk factors for anal cancer in HIV-positive people showed that anal cancer incidence was extensively higher in the ART era as compared to the pre-ART era [115]. This study findings generalized about head and neck cancers; however, a meta-analysis of cancer incidence in people with HIV demonstrated that HIV positive individuals are 2.32 times (95% CI 1.65 to 3.25) as likely to develop oral cavity or pharyngeal cancers as compared to HIV-negative individuals [1].

Despite the limited literature in SSA, in the USA, the estimated cancer risk among HIV-infected individuals as compared to the general population has been estimated to be as high as 29 times increased for anal cancer, 4 times for penile cancer, 6 times for vulvar and vaginal cancer as well as cervical cancer, respectively [1]. The findings of this study demonstrate a need for more research investigating the HPV-related cancer among HIV-infected population in SSA.

### Strengths and limitations of the study

An important strength of this study is that there has been a comprehensive search of relevant studies to be included in this scoping review with no limitation on publication dates and language. This scoping review methodology has allowed for the inclusion of different study designs, and a systematic approach was used to identify relevant studies, charting, and analysis of outcomes [20, 23]. Studies were selected according to the PEO (population, exposure, and outcomes) framework as it was the appropriate heuristic to use based on the framed research question. In addition, the results of the scoping review were presented following the PRISMA recommendation which ensured complete and transparent reporting (Additional file 4: Table S1). Transparent methodological quality assessments of the included primary studies using the recommended MMAT tool assess the risk of bias from the included studies. The study was not limited to a specific population group; this allowed for extensive exploration of HPV-related cancers in PLHIV on different age groups, gender, and different geographical locations in SSA.

Despite the strengths reported for this study, there is a limitation as well. The study was limited with the exclusion of qualitative studies as the research question is more epidemiological, hence the use of PEO framework. Qualitative research was not qualified to be included in this scoping review; therefore, it might have resulted in omission of data.

### Implications for research

This study shows limited evidence on the interaction of HIV and HPV-related cancers especially on anal cancer across all genders, vaginal cancer, vulvar cancer, head and neck cancers, and penile cancer. We hope this study results will prompt further studies to provide better insights on the natural history of HPV-related cancers. More studies to evaluate the risk of developing HPV-related cancers among PLHIV are recommended. Additionally, more future studies to be conducted at different countries within SSA and in rural areas are needed. It also calls for recruitment of more males to participate in research.

### Implications for practice

This study revealed the need for policy makers in public health to take note on the development of HPV-attributable cancers among PLHIV when planning and making decisions, bearing in mind that HIV-infected individuals who live longer on ART may be at increased risk of persistent HPV infection [92]. Our study findings also suggest that HPV infection should be evaluated at various stages of HIV, such as during the initiation of ART and with the long-term use of ART among all infected individuals (men, women, and children). This study’s findings further suggest that more effective control interventions among PLHIV are needed to reduce the burden of HPV-attributable cancers among this group. Moreover, this scoping review findings suggested there were limited research among populations in resource-limited settings; hence, future investigations should consider including populations who live in such settings.

## Conclusion

This study enabled the authors to provide evidence on the interplay of HIV and HPV-related cancers in SSA. Although this study demonstrated shortage of evidence from published studies on the interaction between HIV and HPV-attributable cancers on all identified anatomic sites, high number of studies reported the association of HIV positivity with increased prevalence of HPV infection on different anatomic sites, which will result in increased burden of HPV-related cancers among PLHIV. Therefore, a meta-analysis study is recommended to confirm this association. Furthermore, primary studies with robust study designs aimed at investigating the risk of developing HPV-related cancers among PLHIV in SSA are recommended. Furthermore, primary studies with robust study designs aimed at investigating the risk of developing HPV-related cancers among PLHIV in SSA are recommended.

## Supplementary information

**Additional file 1: Table S1.** Electronic database search results for title screening.

**Additional file 2: Table S1.** Calculation of degree of agreement.

**Additional file 3: Table S1.** MMAT quality assessment tool.

**Additional file 4: Table S1.** PRISMA checklist.

## Data Availability

The data reported and supporting this paper was sourced from the existing literature and are therefore available through the detailed reference list.

## Abbreviations

AIDS: Acquired immunodeficiency syndrome
AIN: Anal intraepithelial neoplasia
ART: Antiretroviral therapy
CIN: Cervical intraepithelial neoplasia
HIV: Human immunodeficiency virus
HPV: Human papillomavirus
Hr-HPV: High-risk HPV
HSIL: High-grade squamous intraepithelial lesion
ICC: Invasive cervical cancer
IARC: International Agency for Research on Cancer
LMIC: Low- and middle-income countries
LSIL: Low-grade squamous intraepithelial lesion
MeSH: Medical Subject Headings
MMAT: Mixed Methods Appraisal Tool
MSM: Men who have sex with men
PLHIV: People living with HIV
PRISMA: Preferred Reporting Items for Systematic Reviews and Meta-Analyses
SSA: Sub-Saharan Africa
WHO: World Health Organization
