# Supplementary material for: The interplay of HIV and human papillomavirus-related cancers in sub-Saharan Africa: scoping review
Source: Syst Rev. 2020 Apr 22;9:88. doi: 10.1186/s13643-020-01354-1 (PMC7178989; doi:10.1186/s13643-020-01354-1)
Supplement: Supplementary file 1 — Additional file 1: Table S1. Electronic database search results for title screening. [file 13643_2020_1354_MOESM1_ESM.docx]

**Additional files**

**Additional file 1**: **Electronic search results**

**Appendix 1: Electronic database search results for title screening**

| **Search date** | **Database** | **Key words** | **Number of studies found** | **Number of studies included** |
| --- | --- | --- | --- | --- |
| 27/07/2017 | Google scholar | Hpv-related cancers prevalence or incidence or trends or mortality or risk factors or HIV or SSA | 4,340 | 90 |
| 27/07/2017 | PubMed | Prevalence or incidence or mortality or risk factors or HIV or SSA AND HPV-related cancers | 8,786 | 44 |
| 26/07/2017 | EBSCOhost platform | Individuals with HPV-related cancers or HPV-related cancers and prevalence and incidence and mortality and trends and risk factors and HIV and SSA | 1,054 | 82 |
| 26/07/2017 | EBSCOhost platform | HPV-related cancer individuals or HPV-related cancers or prevalence or mortality or incidence or trends or risk factors | 448 | 58 |
| 28/07/2017 | Science direct | Prevalence or incidence or mortality or trends or HIV or risk factors or HPV-associated cancers | 11,192 | 22 |
| 29/07/2017 | WHO library | Prevalence or incidence or mortality or trends or HIV or risk factors or HPV-associated cancers | 15 | 01 |
