# Supplementary material for: The interplay of HIV and human papillomavirus-related cancers in sub-Saharan Africa: scoping review
Source: Syst Rev. 2020 Apr 22;9:88. doi: 10.1186/s13643-020-01354-1 (PMC7178989; doi:10.1186/s13643-020-01354-1)
Supplement: Supplementary file 2 — Additional file 2: Table S1. Calculation of degree of agreement. [file 13643_2020_1354_MOESM2_ESM.docx]

**Additional file 2: Calculation of degree of agreement**

**Full article screening results-degree of agreement**

| **Author and date** | **Reviewer 1: response** | **Reviewer 2: response** |
| --- | --- | --- |
| Adelstein, 2010 | No | no |
| Arbyn, 2012 | Yes | no |
| Atlin, 2016 | No | no |
| Auluck, 2010 | No | no |
| Azvolinsky, 2013 | No | no |
| Barroca, 2016 | No | no |
| Barzon, 2014 | No | no |
| Beachler, 2011 | No | no |
| Best, 2010 | No | no |
| Blomberg,2011 | Yes | no |
| Bodelon,2016 | No | no |
| Borget,2011 | No | no |
| Bosch,2002 | No | no |
| Bosch, 2013 | Yes | yes |
| Bosch, 1995 | Yes | yes |
| Brickman, 2015 | Yes | yes |
| Brisson, 2013 | no | no |
| Capo-chichi, 2016 | no | yes |
| Carroll, 2010 | Yes | yes |
| Chaturvedi,2010 | No | No |
| Chaturvedi, 2008 | no | No |
| Chaturvedi, 2009 | No | No |
| Chen, 2004 | No | No |
| Chow, 2015 | No | no |
| Clemente, 2017 | No | no |
| Conway, 2012 | No | no |
| De Sanjosé,2012 | no | yes |
| De Vuyst,2013 | Yes | yes |
| Dillner, 2015 | No | no |
| D’souza, 2007 | No | no |
| Dunne, Eileen F. 2007 | No | no |
| Firnhaber, 2012 | Yes | no |
| Forman, 2012 | Yes | yes |
| Gooi, 2016 | No | yes |
| Grulich, 2007 | No | no |
| Hearda, 2011 | no | yes |
| Jedy-Agba,2016 | Yes | yes |
| Joo, 2013 | no | no |
| Kahn, 2012 | No | no |
| Katz, 2017 | No | no |
| Kreuter, 2009 | No | no |
| Krishnan, 2008 | No | no |
| Levovitz, 2014 | No | no |
| Liu, 2015 | No | no |
| Louie, 2009 | Yes | yes |
| Lowy, 2012 | no | yes |
| Mayeaux, 2008 | No | no |
| Meyer, 2014 | No | no |
| Mirghani, 2017 | No | no |
| Muñoz, 2003 | Yes | yes |
| Natunen, 2011 | Yes | yes |
| Nelson, 2017 | No | no |
| Neumann, 2016 | No | no |
| Nicol, 2011 | No | no |
| Oga, 2016 | Yes | yes |
| Olesen, | Yes | yes |
| Ortiz,2014 | No | no |
| Paavonen, 2007 | No | no |
| Parkin, 2006 | no | yes |
| Schiech, 2010 | No | no |
| Shack, 2014 | No | no |
| Stier, 2015 | No | no |
| Syrjänen, 2011 | No | no |
| Tornesello, 2014 | No | no |
| van der Zee, 2013 | No | no |
| Vivenza, 2016 | No | no |
| Wagner, 2017 | No | no |
| Wakeham, 2014 | No | no |
| O’rorke,2012 | No | no |
| Beachler, 2014 | No | no |
| Palefsky, 2006 | Yes | no |
| Palefsky, 2009 | No | no |
| Palefsky, 1997 | No | no |
| Palefsky, 2017 | No | no |

**The output from STATA**

. kap Reviewer1response Reviewer2response

Expected

Agreement Agreement Kappa Std. Err. Z Prob>Z

-----------------------------------------------------------------

86.49% 64.57% 0.6186 0.1159 5.34 0.0000

. mcc Reviewer1response Reviewer2response

| Controls |

Cases | Exposed Unexposed | Total

-----------------+------------------------+------------

Exposed | 12 4 | 16

Unexposed | 6 52 | 58

-----------------+------------------------+------------

Total | 18 56 | 74

McNemar's chi2(1) = 0.40 Prob > chi2 = 0.5271

Exact McNemar significance probability = 0.7539

Proportion with factor

Cases .2162162

Controls .2432432 [95% Conf. Interval]

--------- --------------------

difference -.027027 -.12407 .0700159

ratio .8888889 .6169294 1.280736

rel. diff. -.0357143 -.148351 .0769224

odds ratio .6666667 .1383716 2.811294 (exact)
