# Supplementary material for: The interplay of HIV and human papillomavirus-related cancers in sub-Saharan Africa: scoping review
Source: Syst Rev. 2020 Apr 22;9:88. doi: 10.1186/s13643-020-01354-1 (PMC7178989; doi:10.1186/s13643-020-01354-1)
Supplement: Supplementary file 3 — Additional file 3: Table S1. MMAT quality assessment tool. [file 13643_2020_1354_MOESM3_ESM.docx]

**Additional 3: Quality assessment table**

**Appendix 3: MMAT Quality Assessment tool**

| Study Type | Methodological Criteria |
| --- | --- |
| Screening questions  (All study types) | - Are there clear research questions/objectives? - Do the collected data address the research question/objective? |
| Quantitative | - Is the sampling strategy relevant to address the quantitative research question? - Is the sample representative of the population under study? - Are measurements appropriate (clear origin, or validity known, or standard instrument)? - Is there an acceptable response rate (60% or above)? |
